# A structural approach reveals how neighbouring C2H2 zinc fingers influence DNA binding specificity

**DOI:** 10.1093/nar/gkv919

**Published:** 2015-09-17

**Authors:** Michael Garton, Hamed S. Najafabadi, Frank W. Schmitges, Ernest Radovani, Timothy R. Hughes, Philip M. Kim

**Affiliations:** 1 Donnelly Centre for Cellular and Biomolecular Research, University of Toronto, Toronto M5S 3E1, Canada; 2 Department of Molecular Genetics, University of Toronto, Toronto M5S 1A8, Canada; 3 Department of Computer Science, University of Toronto, Toronto M5S 2E4, Canada

## Abstract

Development of an accurate protein–DNA recognition code that can predict DNA specificity from protein sequence is a central problem in biology. C_2_H_2_ zinc fingers constitute by far the largest family of DNA binding domains and their binding specificity has been studied intensively. However, despite decades of research, accurate prediction of DNA specificity remains elusive. A major obstacle is thought to be the inability of current methods to account for the influence of neighbouring domains. Here we show that this problem can be addressed using a structural approach: we build structural models for all C_2_H_2_-ZF–DNA complexes with known binding motifs and find six distinct binding modes. Each mode changes the orientation of specificity residues with respect to the DNA, thereby modulating base preference. Most importantly, the structural analysis shows that residues at the domain interface strongly and predictably influence the binding mode, and hence specificity. Accounting for predicted binding mode significantly improves prediction accuracy of predicted motifs. This new insight into the fundamental behaviour of C_2_H_2_-ZFs has implications for both improving the prediction of natural zinc finger-binding sites, and for prioritizing further experiments to complete the code. It also provides a new design feature for zinc finger engineering.

## INTRODUCTION

As the most common protein domain in the human genome, C_2_H_2_ zinc fingers (C_2_H_2_-ZF) are known to espouse a wide variety of roles (1–3), involving the recognition and binding of both nucleic acids and proteins (4–6). DNA binding is likely the most common because auxiliary DNA interacting domains including the potent transcriptional repressors KRAB and BTB (7–9) are often present, and accordingly, most C_2_H_2_ proteins tested by ChIP-seq bind specific DNA sequences (10). C_2_H_2_-ZFs are modular and are connected via short unstructured linkers to form arrays of up to 40 fingers in length. Each finger typically recognizes a triplet of nucleic acid bases (11) and often recognition is limited to a subset of the fingers of an array. The C2H2-ZF DNA binding residues are most commonly defined as four canonical ‘specificity residues’ +6, +3, −1 and +2 located on the α-helix* (although in reality binding is not always limited to these four) (12). Virtually any amino acid can be found at any of the specificity residue positions, and the combination of multiple fingers can achieve remarkable diversity and specificity. Functional description remains elusive for a great majority of the expansive C_2_H_2_ family, although the presence of a KRAB domain in ∼50% of human C_2_H_2_ proteins suggests they are frequently employed in silencing exogenous retroviruses and endogenous retro-elements (13–15).

Determining the DNA binding motif is an important step toward functional characterization and currently only ∼20% of C_2_H_2_-ZF motifs are known (16–18). This is a consequence of the considerable effort required, and unavoidably high rate of experiment failure when trying to determine each motif using methods such as ChIP-seq or protein binding microarrays (PBM). A beguiling alternative is to directly predict DNA sequence preferences from the C_2_H_2_-ZF amino acid sequence (12,19). The challenge to create such a comprehensive ‘recognition code’ is still far from realized despite two decades of research, and the most recent advances allow individual nucleotide prediction with ∼50% accuracy (20,21). Obstacles include: incomplete mapping between specificity residues and base preferences; contribution from amino acids outside of the four specificity residues (22); and the influence of neighbouring C_2_H_2_ domains (23). We recently addressed the first of these issues (10) by determining the DNA sequence preferences of 8138 distinct natural C_2_H_2_-ZFs, sampled from all eukaryotes, using a modified bacterial one-hybrid (B1H) system (24,25). A random forest trained on these data allowed motif prediction that outperformed other recent methods (10). However, many domains still consistently yield poor prediction accuracy regardless of the recognition code used.

**Figure 1.**
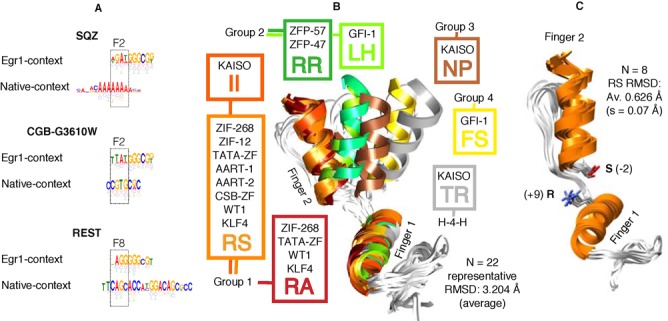
Context-dependent sequence preferences and PDB structural alignments. (**A**) Aligned PWMs showing that the second finger of SQZ, second finger of CGB-G3610W and eighth finger of REST recognize different DNA motifs depending on whether they are in their native context or fused to fingers 1 and 2 of Egr1. (**B**) Alignment of 22 two-finger arrays extracted from 12 non-redundant PDB structures. Showing considerable conformational variation between subgroups and close structural similarity within subgroups. Coloured boxes list the different protein types contributing to structurally similar subgroups together with the identity of residues +9 and −2 that exist at the finger–finger boundary of that group. H-4-H has four rather than the typical three residues separating the two Histidines (**C**) Alignment of all eight 2-finger arrays extracted from the PDB, where residues +9 and −2 are Arg and Ser respectively. Structural similarity between any two arrays is <1.0 Å Cα RMSD despite seven different proteins contributing to this subgroup.

A potential shortcoming in the derivation and use of most recognition codes is that the influence of native context adjacent domains is not accounted for. Influencing factors are thought to include domains sharing a base pair—known as the *subsite overlap* (23,26,27), and different combinations of specificity residues on neighbouring domains (28–30). The precise nature of neighbour influence remains enigmatic however, highlighted most recently by investigation of yeast C2H2-ZF, which reported widespread differences in DNA binding preferences among fingers with identical DNA specificity residues (31). Examples illustrating consequences of the neighbour context problem are shown in Figure 1A. Motifs preferred by the second finger of SQZ, second finger of CGB-G3610W and eighth finger of REST—in their native context—are very different from those preferred by the same domain fused to fingers one and two of the classic Zif268 array (as was the context in B1H experiments(10)).

To investigate the problem of neighbour influence from a structural perspective, we examine different binding conformations among the available PDB C_2_H_2_ structures and expand these using molecular modelling to access all binding conformations extant in a gold standard C_2_H_2_-ZF—DNA dataset (see ‘Materials and Methods’ section). Surprisingly, our analysis indicates that residues at the protein–protein interface between adjacent domains appear to constrain the relative orientation of adjacent domains. We propose that these constraints in orientation allow or disallow certain residue—base contacts, giving rise to the neighbour effect problem. The resulting set of ‘binding modes’ informs a solution to the problem of neighbour effects across a large proportion of putative DNA binding C_2_H_2_-ZFs.

## MATERIALS AND METHODS

### Protein database analysis

All 39 C2H2-ZF protein DNA complexes were retrieved from the protein database (PDB). Redundant structures, or those with <3 Å resolution were discarded reducing the number to 11 (2DRP, 2I13, 2KMK, 4F6M, 1A1F, 1G2F, 2PRT, 4M9E, 4GZN, 4M9V, 1F2I, 1MEY). Every possible two-finger array was extracted from these 12 structures and after removing those disassociated from their nucleic acid (shortest ZF—DNA distance >10 Å), a set of 22 structures remained. Structures were visualized using Chimera and aligned using the first finger of each two-finger set to highlight relative differences in finger 2 position. Sequence alignments performed using ClustalX.

### Molecular modelling

Structural models were generated with the Jackal (32) program using PDB file 1AAY as the template. The DNA was elongated by 4 bp at each end using X3DNA (33) such that any end effects (termini melting) would not affect the protein bound nucleotides. Zinc 2+ ions were approximated using four mass-less dummy atoms in addition to the core zinc, each with a 1/2 positive charge, in a tetrahedral arrangement, for correctly orientated coordination of the four C_2_H_2_-ZF side-chains (34). One model was made for every possible three-finger array using a 36-member subset of the gold standard set. The DNA component was mutated in each case using Chimera (35) to produce the sequence determined by experiment (B1H, PBM or ChIP-seq) for that C_2_H_2_-ZF array. All models were prepared for Amber MD simulation using the WHATIF web interface (36) to build in any missing atoms and identify protonation states. They were then explicitly solvated in a 10 nm3 box of TIP3P water using TLEAP in AMBER 10 (37). Sodium counter-ions were added for overall charge neutrality and periodic boundary conditions were applied. Bonds to hydrogen were constrained using SHAKE (38) to permit a 2 fs time step and the particle mesh Ewald (39) algorithm was used to treat long-range electrostatic interactions. The non-bonded cut-off was set at 12.0 Å. Systems were energy minimized using a combination of steepest descent and conjugate gradient methods. MD calculations were carried out with the PMEMD module of AMBER 10 in conjunction with the FF99 Barcelona forcefield (40), which is specifically customized for nucleic acids. The FF99 Stony Brook forcefield (41) was used for the protein. Each system was equilibrated and heated over 100 ps to 300K and positional restraints were gradually removed. A Berendsen thermostat and barostat was used throughout for both temperature and pressure regulation (42). A total of 20 ns of conformational space exploration was obtained for each array. During calculations, a snapshot was saved every 2 ps. Root mean square deviation (RMSD) was evaluated to assess the equilibration of each run. RMS clustering of the trajectory frames was carried out using the MMTSB toolset (43) kclust, with the radius set to 2.5 Å and maxerr to 1. This produced a set of 45 representative conformations. Further Jackal modelling was then carried out using these conformations as the templates. Every possible three-fingered array in the full gold standard set, together with their cognate motifs, were committed to each template, producing 90 models. The exact register of fingers and specificity residues with respect to the known motifs—where not obvious—was determined using ChIP-seq enrichment data and recognition code predictions where agreement allowed.

### Gold standard set compilation

We compiled a set of 64 ‘gold standard’ motifs from the literature and available databases for natural C2H2-ZF proteins from different organisms. We used the collection of motifs reported for C2H2-ZF proteins in the CisBP database (44), including only C2H2-ZF proteins with canonical linker lengths (4–6 amino acids). A single motif for each model was selected—to obtain a manageable number of models for MD simulation—by removing redundant motifs. For each protein with multiple motifs, we first selected a single representative motif as follows: if the protein had more than two motifs, we selected the motif that had the largest ‘sum of similarities’ to other motifs. The similarity of a pair of motifs was defined as the Pearson correlation of their affinity scores across 50 000 random sequences of length 100 bp, with affinity scores calculated as described previously (45). If the protein had only two motifs but also had a characterized homolog, we selected the motif that was most similar to the homologue motif (reasoning that this motif was supported by an independent experiment from a similar protein). If the protein had only two motifs and no homologues, we selected one motif randomly. We further removed similar motifs from different proteins by performing Affinity Propagation clustering of the motifs (46), selecting only the ‘exemplar’ motif from each cluster. In some cases the precise residue—base register was difficult to determine, for instance, where an experimentally determined motif is longer than necessary to accommodate an array. Such cases were excluded to optimize accuracy, leaving a high accuracy subset of 36 cases for modelling and preference profile determination. The 28 remaining cases were retained as a test set for motif prediction using a random forest.

### Preference profiles

Preference profiles were produced using the high accuracy subset of 36 arrays discussed in the previous section. These 36 arrays were split into their constituent fingers and each finger associated with its boundary pair identifier, comprising residue +9 of the previous finger and −2 of the subject finger. Fingers were further associated with a position weight matrix (PWM) delineating experimentally determined nucleotide preferences for that domain. Precise residue—base associations could be determined because all 36 PWMs contained the same number of consecutive base triplets as the fingers of their associated array. Mean frequencies were calculated for each of the four specificity residues (+6 +3 −1 +2) across the entire gold standard set to produce a preference profile that does not account for binding mode influence. Fingers were then grouped according to their boundary pair association with one of six structural modes determined from the crystal structures and MD models. Mean frequencies were calculated for each of the four specificity residues across each of the six separated groups to produce preference profiles that reflect the influence of binding modes. Amino acids present in the gold standard set for each specificity position were ordered from left to right according to decreasing incidence. The frequency information for each amino acid was used to calculate the statistical significance of these differences and p-values reflecting this were plotted above each profile.

### Introducing modes into the specificity code

Machine learning calculations were carried out using the RandomForest R package with a model composed of 2000 decision trees. The random forest was trained on binding preferences from the gold standard 36-member subset (see above). Predictions with response type output were made using 10-fold cross validation and also of the 28-member gold standard subset. Predictions were aligned to known motifs to identify the register for the 28-member subset. Training was carried out without mode information using all 100 fingers with four specificity residues as covariates and correction bias applied. This was repeated 16 times to get each of the 16 (4 × ACGT) base preferences and capture all specificity residue influence for the full set. To involve modes, the process was repeated including residue +9 from the previous finger and −2 of the subject finger as covariates, making six features in total. Where amino acids did not exist in the training set (i.e. empty positions in Figure 3) recognition code (10) predictions were inserted. Both sets of predictions were aligned to experimental motifs (10-fold cross validation and the 28-member test set) and similarities were calculated using Pearson correlation.

### Correlating structural similarity with boundary pairs and modes

All 108 structures from the PDB and modelling were aligned with every other to produce a matrix of 11 664 RMSD measurements. To avoid cases where conserved boundary pairs simply reflected the close overall sequence identity of an array, pairs of structures with a sequence identity of >50% were removed. Remaining measurements were grouped according to whether or not the pair of structures had matching +9/−2 boundary pair residues. Conformational difference is strongly correlated with boundary pair match/mismatch.

## RESULTS

### PDB structures reveal boundary residue-specific conformations

To investigate the influence of immediately neighbouring finger domains on a particular C_2_H_2_-ZF, we first examined all available crystal and nuclear magnetic resonance (NMR) structures extant in the PDB. After discarding structures with low resolution (>3.0 Å) and poor DNA association (shortest α-helix—DNA distance >10.0 Å), we extracted and examined 22 non-redundant pairs of adjacent zinc fingers from these structures. In the process of this analysis, we noted that any two adjacent fingers appear to adopt one of a small number of relative orientations. Figure 1B shows that there is significant conformational variation between these arrays but that some cluster very closely, with Cα RMSD <1.0 Å. This similarity is despite the structures originating from different proteins and involving considerable sequence difference (Supplementary Figure S1). Remarkably, we found that when grouped according to structural similarity (defined as Cα RMSD of <1.0 Å), arrays within each group often have conserved residues at positions +9 from ‘finger 1’ and −2 from ‘finger 2’ (fingers are oriented from N to C termini). These are residues that interact at the finger—finger boundary. Other residues that could potentially be involved in boundary interactions—including those involved in DNA readout at the sub-site overlap(25,26)—were not conserved within these conformational groups. Conservation can't be explained by common ancestry as in many cases the proteins within structural groups are not homologues. An illustration is shown in Figure 1C where a group of nine different two fingered arrays from seven different proteins all align with an RMSD of 0.6 Å. In this case conserved residue positions +9 and −2 are Arginine and Serine, and this combination is not seen in any other group. Thus, while formation of energetically favourable finger-DNA contacts during the binding process undoubtedly influences domain orientation to a certain extent, specific residues at the interface between the two domains appear to play a dominant role.

### Expanding the structure set using MD simulation

**Figure 2.**
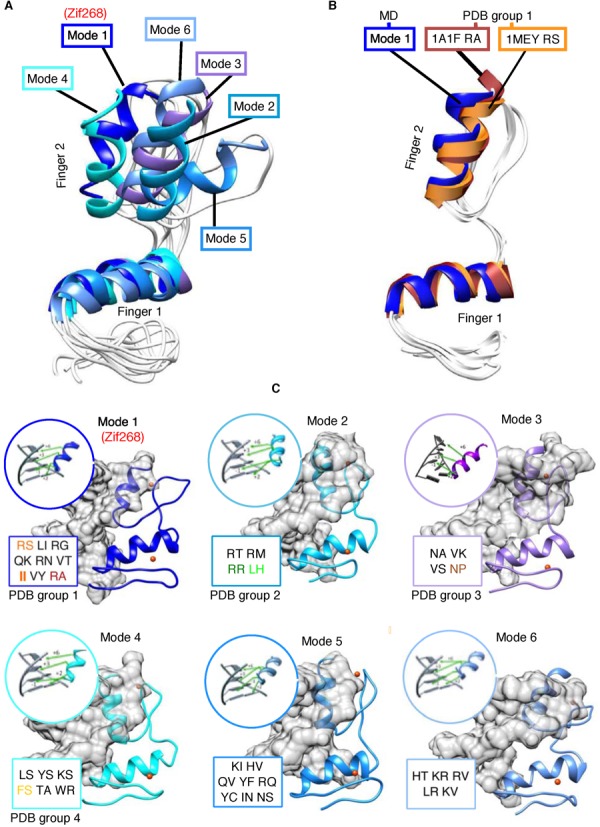
Binding modes with associated boundary pairs and specificity residue orientations. (**A**) Alignment of structures representing six binding modes identified by clustering MD trajectory snapshots. Superimposing produces a minimum of 2.0 Å Cα RMSD between any two structures. (**B**) Representative structure for mode 1, aligned with two structures from the PDB (1A1F and 1MEY) that are representative of the RA and RS boundary pair contribution to the most highly populated structural group from PDB analysis (**C**) Differences in orientation of finger 2 and its canonical specificity residues with respect to the major groove for each mode. Variation in optimal hydrogen bond geometry influences base preference. Boundary pairs relating to each mode are shown inset, with those captured by PDB structures colour-coded according to Figure 1.

The paucity of C_2_H_2_ structures makes it unlikely that all possible conformational variants have been captured. We therefore next employed molecular modelling to expand structural space to include all C_2_H_2_–DNA complex conformations where the motif has been experimentally determined with high confidence. Models were constructed for every possible three-finger sub-array in a gold standard set subset (see ‘Materials and Methods’ section) of 36 C_2_H_2_-ZFs that had varying lengths of 3–10 zinc fingers, adding up to a total of 90 three-finger sub-arrays. Each was in complex with an 18 bp DNA fragment bearing the experimentally determined base sequence motif for that array. MD simulation in explicit solvent, followed by clustering of the output equilibrated trajectories produced representative conformations for each complex. RMSD alignment of the conformations revealed extensive conformational redundancy. When grouped along with PDB structures, six distinct conformational clusters were observed, which we refer to hereafter as binding ‘modes’. Figure 2A shows a structural alignment of the representative structures for each mode. Structural similarity within each mode was <1.5 Å Cα RMSD, while each mode was distinguished from the others by containing no member with <2.0 Å similarity to any member of another mode. Alignment between the modelled structures and MD relaxed PDB structures, showed they adopt the same modes and intra-mode conformational variation. For example, Figure 2B shows that the mode 1 representative structure aligns closely (<1.0 Å Cα RMSD) with PDB structure Group 1 from Figure 1. Other PDB–mode alignments are shown in Supplementary Figure S2. In a similar manner to the PDB structures, each structurally similar group had residues at position +9 of finger 1 and −2 of finger 2 that were exclusive to—and repeated within—each group. It should be emphasized at this point that modes 5 and 6 are not represented in the PDB and thus do not yet have experimental confirmation for their existence. These results support the notion that, to a first approximation, these residues specify one of six binding modes.

### Each mode has a distinctive base preference profile

We hypothesized that these discrete conformational differences may influence DNA sequence preference. Because each binding mode exhibits a different α-helix orientation in the DNA major groove, the six modes each produce characteristically different canonical residue angles and distances with respect to DNA bases (Figure 2C). Some of this variation can be compensated for by changes in DNA morphology (double helix bending and twisting), however the comparative rigidity of DNA precludes full compensation. Consequently, optimal interacting group geometry varies—allowing for the prospect that a different base could be more energetically favourable to binding by the same side-chain type. An example of this may be found by comparing the solved structures 1a1f and 2kmk (Supplementary Figure S3), where a threonine at the +6 position in both structures associates with thymine on opposite DNA strands, according to the different helix orientation engendered by Arg-Ser and Phe-Ser respectively.

**Figure 3.**
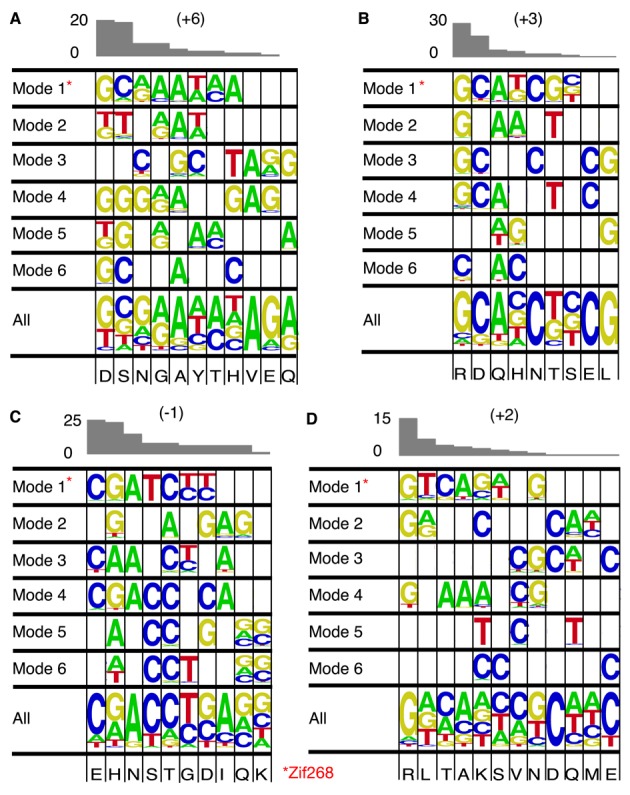
Residue—base preference profiling. (**A**–**D**) Base preferences of each specificity residue at each of the specificity positions +6 +3 −2 +1. Res +2 preferences are shown as the complement base to the actual contact (which is typically cross-stranded). Preferences are shown for all fingers in the 36-member gold standard subset (labelled ALL) and also for fingers grouped into each of their six modes. Empty squares reflect interactions not captured in the gold standard set.

To ask whether residue–base preferences are modulated by neighbouring domains, base preferences were gleaned from the 36-member gold standard subset for the four canonical C_2_H_2_-ZF DNA binding residues of each finger. A profile for each mode was produced (see ‘Materials and Methods’ section) that details the nucleic acid base preference for each of the 20 amino acids at each of the canonical positions: +6, +3, +2 and −1 (Figure 3A–D). Known individual specificity residue preferences from the gold standard set were grouped according to the mode of the finger bearing them. A profile was also generated for the entire set without mode separation (labelled ‘ALL’). Amino acid incidence in the gold standard set is shown above each profile as an indicator of reliability. Gold standard set cases where the precise residue—base register was difficult to determine, were not used, to promote accuracy (e.g., where an experimentally determined motif is longer than necessary to accommodate an array).

Without mode separation, the base preference information of a given specificity residue often includes two or more base types of indistinguishable preference (as observed in the original specificity code (47)). Once the information is separated into modes, however, distinct preference for one specific base can be attributed to many residues and accordingly preferences often differed between modes. There are many specificity residues for which the preference between two bases remains unclear. This is largely resolved by considering both 3′ and 5′ adjacent fingers and therefore four boundary pair residues. There is little benefit from this however, because requiring a combination of 2 modes/4 residues for each finger vastly reduces the information at each specificity position. The influence of more distant neighbour effects may also affect an ability to resolve individual base preferences, as some mode effects may propagate along an array. Some residue—base preferences are less sensitive to change in domain orientation. For example Arginine almost always recognizes Guanine irrespective of mode or position. Long flexible residues such as Arginine can adopt a myriad of rotamers allowing them preserve optimal residue—base geometry.

### Predicting binding mode from sequence using boundary residues +9 and −2

We reasoned that knowledge of different binding modes could be exploited for motif prediction purposes if the modes could be determined from amino acid sequence alone. In essence, the results in Figure 3 suggest that a separate recognition code could be derived for each of the six modes. Because the most preferred motif sequence is unknown *a priori*, MD cannot easily determine binding modes. Predicted motifs could perhaps be used, although the high computational cost for each MD simulation makes it impractical for large-scale investigation, especially given the C_2_H_2_ family size.

**Figure 4.**
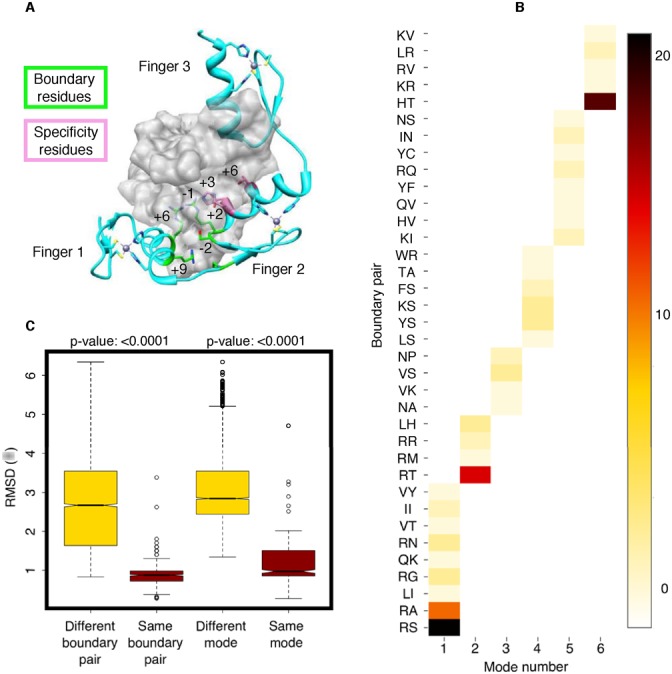
Depiction of boundary residues, heatmap of the modes they are associated with, and correlation of these with structural similarity. (**A**) Zinc finger array in complex with DNA showing the specificity residues +6, +3, −1 and +2. Residues that interact at the boundary between two adjacent fingers are shown. (**B**) Heat map detailing which interacting boundary pairs are responsible for each of the six modes. No pair is associated with more than one mode and intensity indicates the frequency of occurrence for each pair in the gold standard set. (**C**) RMSD alignment of 108 structures from the PDB and modelling. Measurements grouped according to whether or not the pair of structures had matching +9/−2 boundary pair residues, or had the same or different mode. Conformational difference is strongly correlated with boundary pair and mode match/mismatch.

*A priori*, of particular interest for prediction were the non-linker residues of one finger that interact with non-linker residues of a neighbouring finger, especially given the observed sequence conservation of positions +9 and −2. Figure 4A shows four residues (coloured green) that commonly interact at the boundary (finger 1 positions +6 and +9 with finger 2 positions −1 and −2). Finger 1 position +9 also interacts with a linker residue. Modulation of the mode may involve all of these residues and thus be dependent on thousands of combinations in human zinc fingers alone. Gold standard set coverage currently only extends to ∼100 of these combinations, and for this reason, prediction using the minimum possible number of residues is desirable to boost coverage. We explored predicting binding modes by sequence similarity clustering using: full-length finger-linker-finger sequence, all boundary residues, and various other subsets. Neither full sequences nor any of the subsets improved on simply using +9 and −2 to determine modes.

The power of using +9/−2 for prediction is demonstrated by aligning all of the 108 structures produced by MD relaxation of structures from the PDB and modelling with each other, to produce a matrix of 11 664 RMSD measurements. To avoid cases where conserved boundary pairs simply reflected the close overall sequence identity of an array, pairs of structures with a sequence identity of >50% were removed. By plotting each remaining measurement according to whether or not it derived from a pair of structures with matching +9/−2 boundary pair residues (Figure 4B), it can be seen that conformational difference is strongly correlated with boundary pair match/mismatch. Each mode contains different boundary pairs and these are exclusive to that mode—yet often observed multiple times within it (Figure 4C). Plotting structural similarity according to whether or not the pair of structures are included in the same boundary pair determined mode, confirms the power to predict modes using +9/−2 residues.

**Figure 5.**
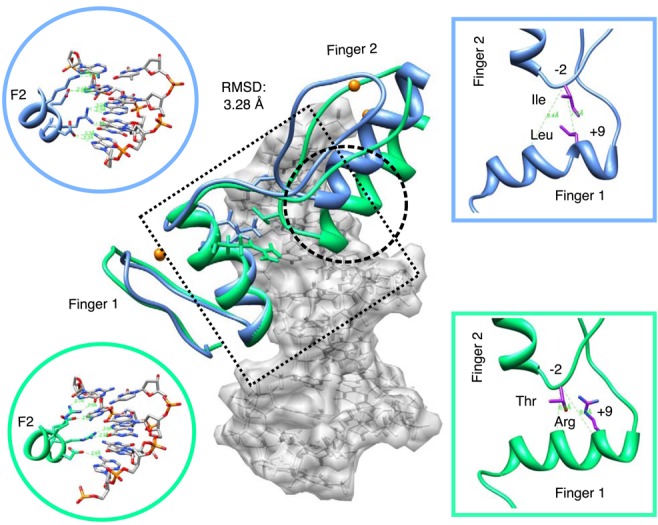
Example of boundary pair geometry differences. Hydrophobic pair (blue) LI and polar pair (green) RT have different spatial arrangements that modulate finger two orientation with respect to DNA. Different finger orientation alters the way that specificity residues are positioned with respect their cognate bases, accounting for differing preferences (circular inset).

The boundary pair effect makes sense structurally as different +9/−2 residue pairings are forced to adopt different positions with respect to each other according to their bulkiness (VdW), electrostatic interactions and hydrophobic effects. These constraints can in turn impose characteristic restrictions on the binding orientations an entire domain can adopt—thus engendering different modes. The models suggest that each pair uses either hydrophobic patch formation or optimal positioning of polar groups to provide significant variation in boundary geometry. Figure 5 shows an example from the modelled set of two distinct pair interactions and the ramifications for finger orientation and hydrogen bond geometry. Compared to R-T residue pairs at +9/−2 positions, the hydrophobic patch adopted by the L-I pair is predicted by the model to induce a 3.1 Å shift and 15° change in angle of finger B with respect to the DNA major groove. Crystal and NMR structures in Supplementary Figure S3 show that threonine prefers thymine on opposite strands, depending on α-helix orientation.

### Using boundary pairs to improve motif prediction

**Figure 6.**
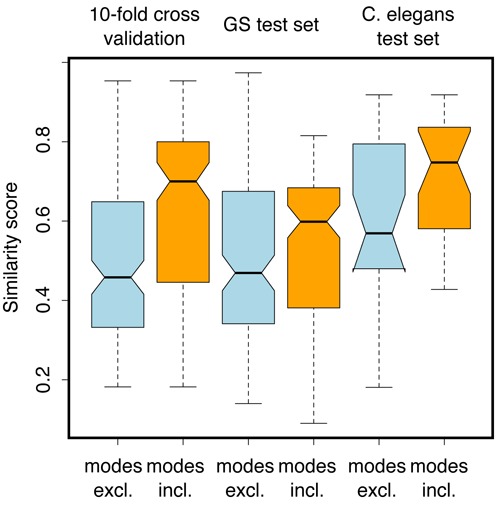
Using boundary pair information to improve random forest predictions. Plots showing the similarity of random forest predicted motifs to those from experiment. Training on the 36 gold standard subset preferences with boundary pair information allows improved prediction under both 10-fold cross validation (left) and a 28-member test set (right).

A random forest was trained on a high accuracy subset (see ‘Materials and Methods’ section) of the gold standard set residue–base preferences. First with the four specificity residues as covariates, followed by training on these—plus the relevant boundary pair residues. Figure 6 shows that preference prediction under 10-fold cross validation was improved significantly by the involvement of boundary pairs (*P*-value < 0.001) Test set prediction of the 28 gold standard set cases—originally discarded due to having indeterminable register—also showed a significant improvement with the inclusion of boundary pair information (*P*-value < 0.001). To ensure that our structural approach is not just fixing idiosyncratic errors in this particular RF model, we used alternative recognition codes developed by Singh *et al*. (20) and Stormo *et al*. (21). We find that our approach leads to a significant improvement for the Singh code (*P*-value < 0.043) and moderate improvement for the Stormo code (*P*-value < 0.09). This suggests that our approach indeed leads to a general solution of this aspect of the neighbourhood problem (Supplementary Figure S5). A very recently published *Caenorhabditis elegans* dataset (48) containing 35 arrays that are not homologous with any gold standard set members was used as an additional benchmark. The inclusion of boundary pair information to correctly predict the PBM results was again beneficial (*P*-value: 0.0379).

## DISCUSSION

We suggest a structural approach to the problem of context dependence on C_2_H_2_-ZF DNA recognition. The existence of a range of distinct C_2_H_2_-ZF binding modes, effected by variation in protein sequence dependent inter–finger interactions, engenders different specificity residue orientation and consequently base preference. Such insight into the fundamental behaviour of this, the largest family of TFs, facilitates a significant improvement in prediction accuracy. The modulating effect of binding modes may also contribute to the explanation for DNA binding behaviour by yeast zinc finger proteins, where widespread differences in specificity were observed for 2-ZF proteins with identical DNA specificity residues (31). Siggers *et al*. proposed that these fingers recognize diverse DNA motifs because the DNA sequence itself engenders different ZF conformational modes, but they suggest that other mechanisms are also operating. As these proteins bind to diverse DNA motifs according to boundary pair mode binding preferences (Supplementary Figure S6), it is likely that the full ZF specificity range is achieved through a combination of influences both from the DNA sequence and from inter-domain residue—residue contacts.

One consequence of different modes may be that some decrease the specificity of a finger. The data in Supplementary Figure S6 shows possible examples of this, where YPO22, CRZ11 and CRZ1 all appear to exhibit reduced specificity compared to others with the same specificity residues. Hypothetically, there might be instances where this is evolutionarily favourable. Even if rendered null—incapable of making productive DNA contacts—a finger would still influence binding preference of the array by controlling the orientation and register of its neighbours. In theory, this might expand the accessible specificity space of ZFs and be an explanation for any specificity variation that has yet to be accounted for.

The importance of neighbour effects has been widely discussed (10,23). Despite this, relatively few structural studies(49) have probed further than the Zif268 model, and with residue variation limited to finger 3, little difference in conformation was captured. Knowledge of the role of modulating boundary pairs may be useful to guide future experimental determination of motifs. By concentrating on the most frequently occurring boundary pairs in nature that are not yet represented or are under represented in the gold standard set, they may—if determined for a few—facilitate better predictions for many. A similar argument holds for generating datasets with the aim of fully populating the tables shown in Figure 3. Most commercially available zinc fingers are either mode 1 or 4 (50) so a complete set of predictions for these modes would be useful for zinc finger nuclease design.

The number of potential boundary pairs is large (400), but only relatively few of them occur frequently. For example, the 39 different boundary pair combinations available in the gold standard set all together cover 73% of all of the human zinc fingers (and their N-terminal neighbour). A total of 239 boundary pairs exist in the human set but the top 25 most frequently occurring account for ∼80% of fingers (Supplementary Figure S4). While many of these are represented in the gold standard set, some are noticeably absent and the future addition of arrays containing these boundary pairs to the gold standard set would lead to significant coverage increase. Coverage is also limited by non-complete residue—base preference profiles for each mode (Figure 3), meaning that in many cases individual base predictions are missing. Our results hence guide future experiments for the most effective completion of the recognition code.

The lack of access to complete coverage when using an approach dependent on the information from limited published motifs means that it is most effectively used to augment other recognition code methods. Replacement can occur when there is a lack of specificity at a given nucleotide position in the motif (Supplementary Figure S7 for set of improved logos selected at random). Making corrections informed by mode preferences to predictions from the latest recognition codes significantly improves prediction accuracy. Combination allows full coverage and optimal accuracy to be achieved.

Our results have implications for both predicting natural zinc finger-binding sites, and the rational design of novel arrays to target motifs of therapeutic or other interest. Further work is needed to clearly elucidate the role—if any—these boundary residues played in the evolutionary history of DNA binding diversification. Modulating boundary pairs may also be interesting from an evolutionary perspective because a single substitution could potentially alter all of the specificity residue base preferences of a DNA binding domain at once, thus providing yet another level of plasticity for the ubiquitous C2H2 domain.

## Supplementary Material

SUPPLEMENTARY DATA
