# Supplementary material for: A structural approach reveals how neighbouring C2H2 zinc fingers influence DNA binding specificity
Source: Nucleic Acids Res. 2015 Sep 17;43(19):9147–57. doi: 10.1093/nar/gkv919 (PMC4627083; doi:10.1093/nar/gkv919)
Supplement: SUPPLEMENTARY DATA [file supp_43_19_9147__index.html]

A structural approach reveals how neighbouring C2H2 zinc fingers influence DNA binding specificity — A structural approach reveals how neighbouring C2H2 zinc fingers influence DNA binding specificity — SUPPLEMENTARY DATA 

# A structural approach reveals how neighbouring C2H2 zinc fingers influence DNA binding specificity

## SUPPLEMENTARY DATA

- SUPPLEMENTARY DATA
