# Supplementary material for: A structural approach reveals how neighbouring C2H2 zinc fingers influence DNA binding specificity
Source: Nucleic Acids Res. 2015 Sep 17;43(19):9147–57. doi: 10.1093/nar/gkv919 (PMC4627083; doi:10.1093/nar/gkv919)

Supplementary Figures for "Breaking the C2H2 recognition code enigma: a structural approach reveals the influence of neighbour context on DNA binding specificity"

<u>Table of contents</u>

Figure S1. Sequence alignments of all non-redundant C2H2 two-finger sets from the PDB.

Figure S2. Representative structures for modes 2 and 6 aligned with PDB structures of similar relative conformation.

Figure S3. Crystal (1a1f) and NMR (2kmk) structures.

Figure S4. Top 25 most frequently occurring boundary pairs (finger 1 residue +9 and finger 2 residue -2) in all human C2H2 zinc fingers.

Figure S5. Box plot showing third party prediction web server prediction improvement.

Figure S6. DNA specificity preferences for groups of yeast 2-ZF's with identical specificity residues and different boundary pairs.

Figure S7. Improved recognition code logos, randomly selected

Figure S8. Box plot showing the range of similarities between the best alignments of model motifs with HT-SELEX produced motifs

Figure S1. Sequence alignments of all non-redundant C2H2 two-finger sets from the PDB. Comparative difference in sequence can result in close structural similarity, providing that residues +9 of finger one and -2 of finger two are the same, for example: #14 and #15. Conversely, comparatively better overall sequence similarity can result in significant differences in relative conformation when this pair of residues differs between structures, for example: #8 and #20.

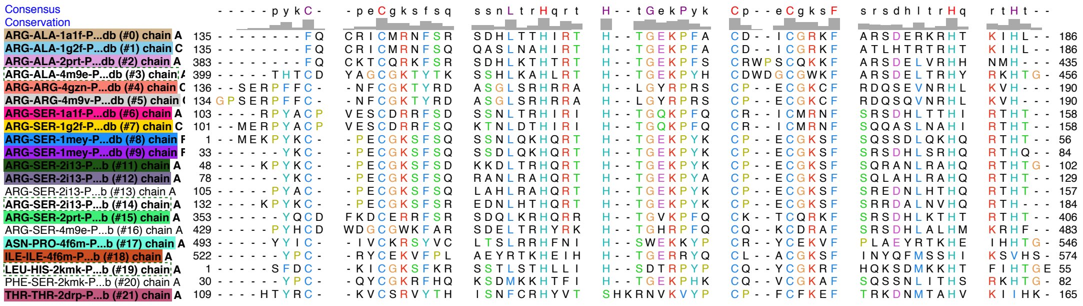

Figure S2. Representative structures for modes 2 and 6 aligned with PDB structures of similar relative conformation. (**Left**) Representative structure for mode 2 aligned with 4 structures from the PDB (4GZN, 4M9V, 2KMK, 4F6M) representing the RR, LH and II boundary pair contribution to the second most highly populated structural group from PDB analysis. (**Middle**) representative structure for mode 5 aligned with a single structure from the PDB (2KMK) involving the FS boundary pair. (**Right**) representative structure for mode 6 aligned with a single structure from the PDB (4F6M) involving the NP boundary pair. In all three cases the RMSD is <1.5 Å between any 2 structures in a group.

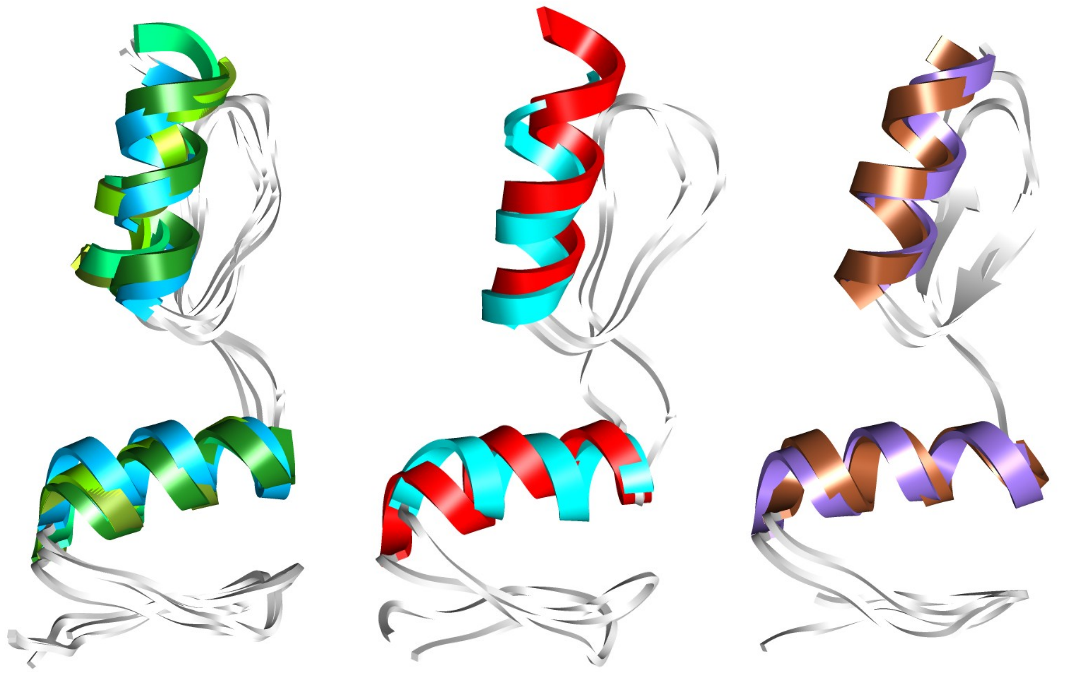

Figure S3. Crystal (1a1f) and NMR (2kmk) structures. Showing that threonine at the +6 position in both structures prefers thymine on opposite strands, depending on the differing α-helix orientation engendered by RS and FS.

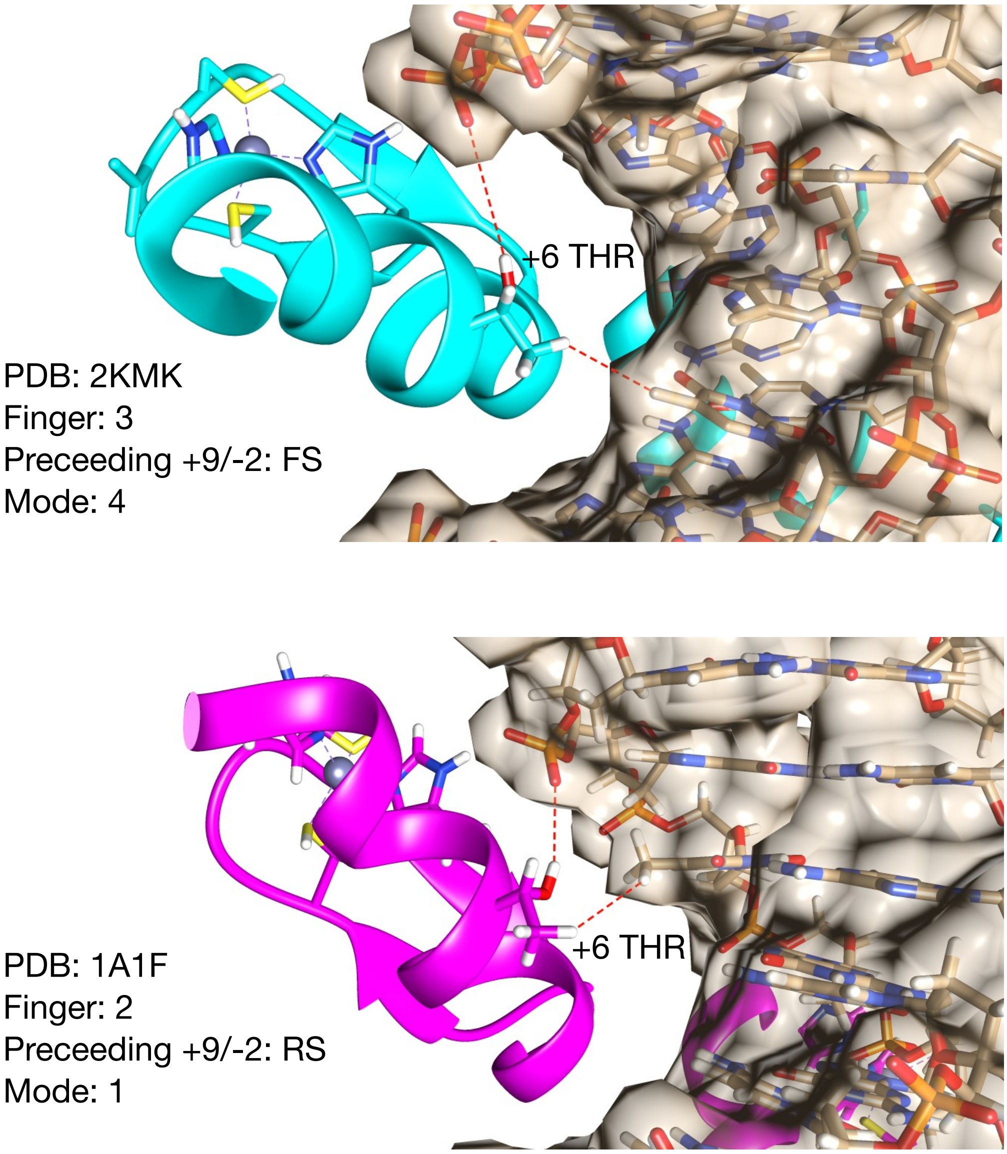

Figure S4. Top 25 most frequently occurring boundary pairs (finger 1 residue +9 and finger 2 residue -2) in all human C2H2 zinc fingers. Boundary pairs also available in the gold standard set are marked in red, while those marked in blue are missing from the set. The most common boundary pairs in humans are shown on the X-axis and total occurrences on the Y-axis. The most common boundary pair is RS with 1306 instances.

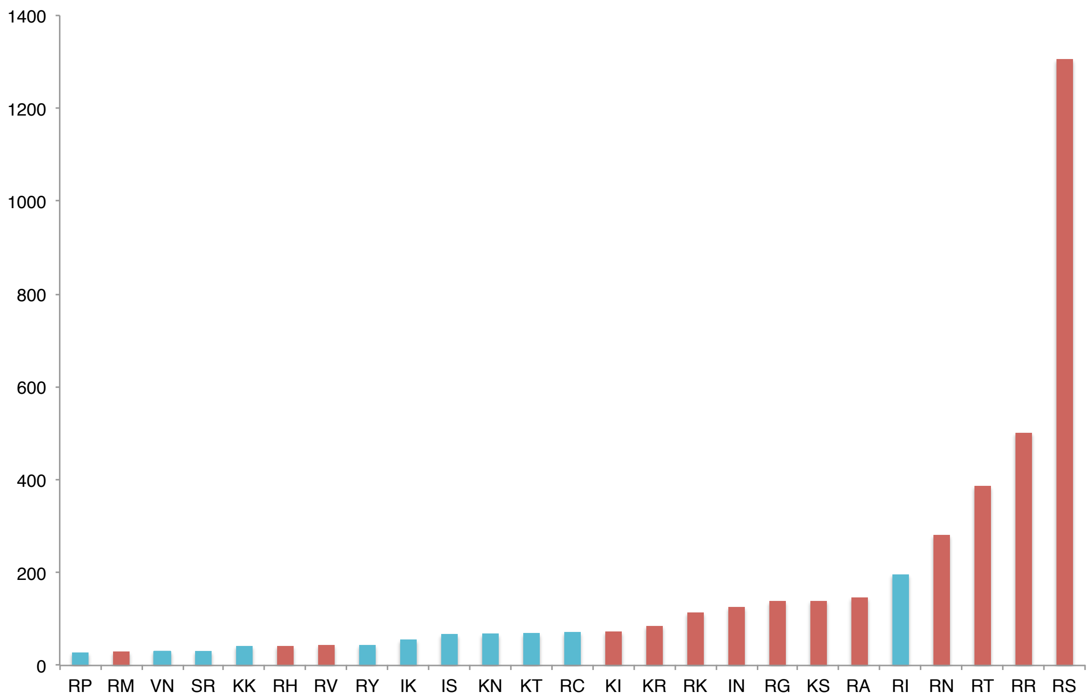

Figure S5. **Box plot showing third party predictions improvement.** (p-values: 0.0431 & 0.0903 respectively)

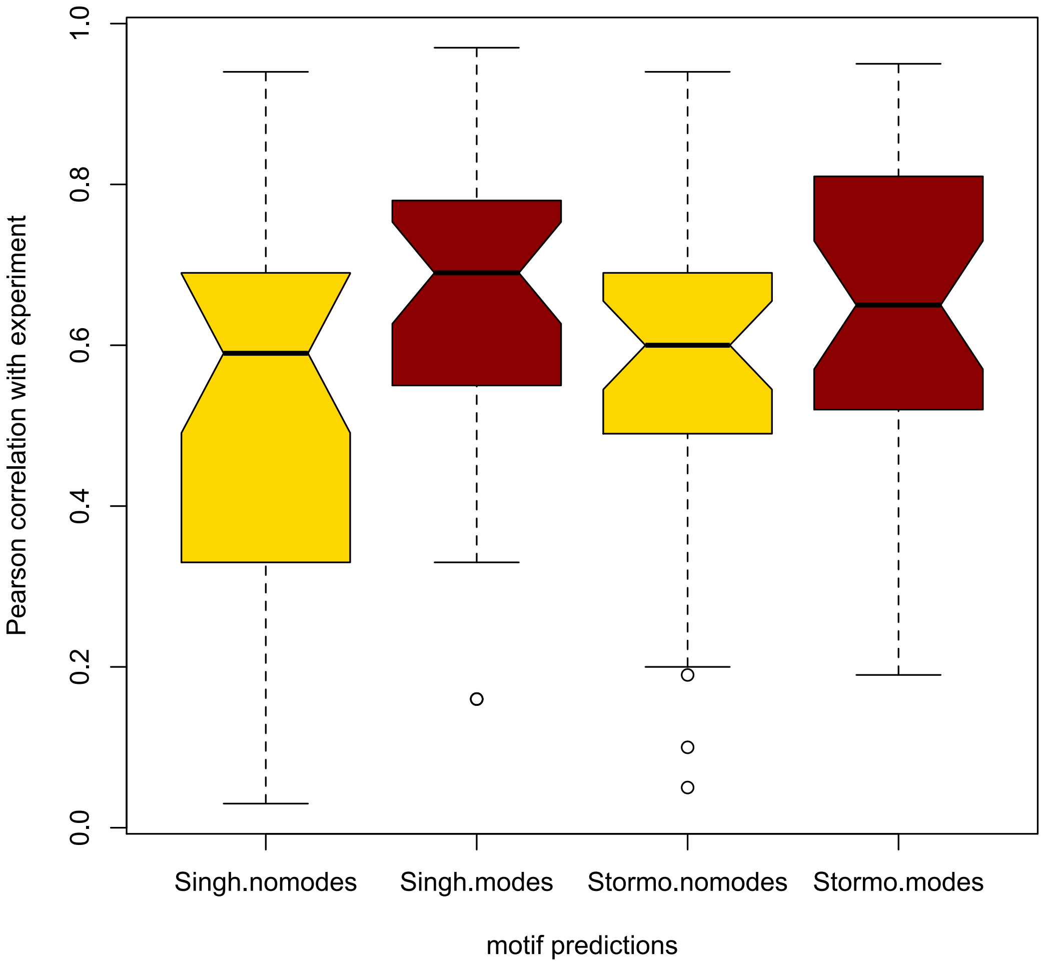

Figure S6. **DNA specificity preferences for groups of yeast 2-ZF's with identical specificity residues (blue squares) and different boundary pairs (red dots).** The binding motif for each set is shown below each group alignment with boundary pair identities in parentheses. (A) Group of five 2-ZFs with specificity residues: REHR-RDLR. (B) Group of three 2-ZFs with specificity residues: RYNS-RHDR. (C) Group of two 2-ZFs with specificity residues: RNDR-RDAR.

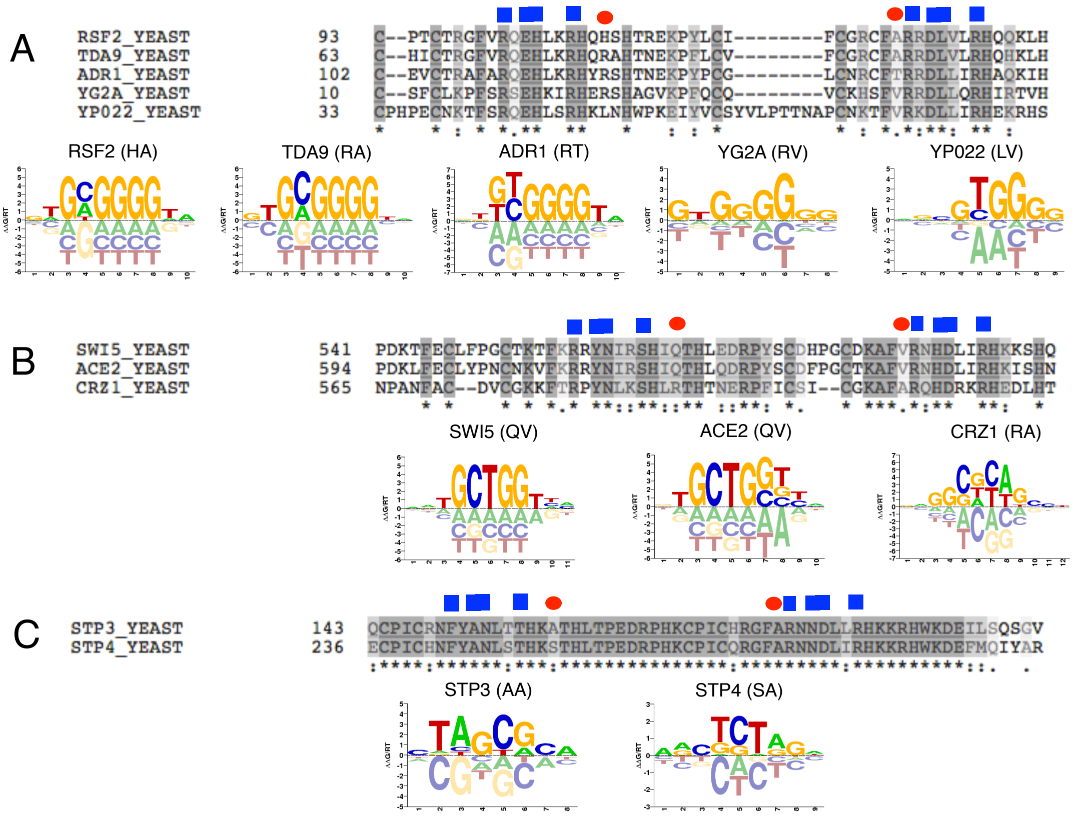

Figure S7. Improved recognition code logos, randomly selected. EXP = experimentally determined motif, RCP = recognition code predictions, MCRC = mode corrected recognition code predictions.

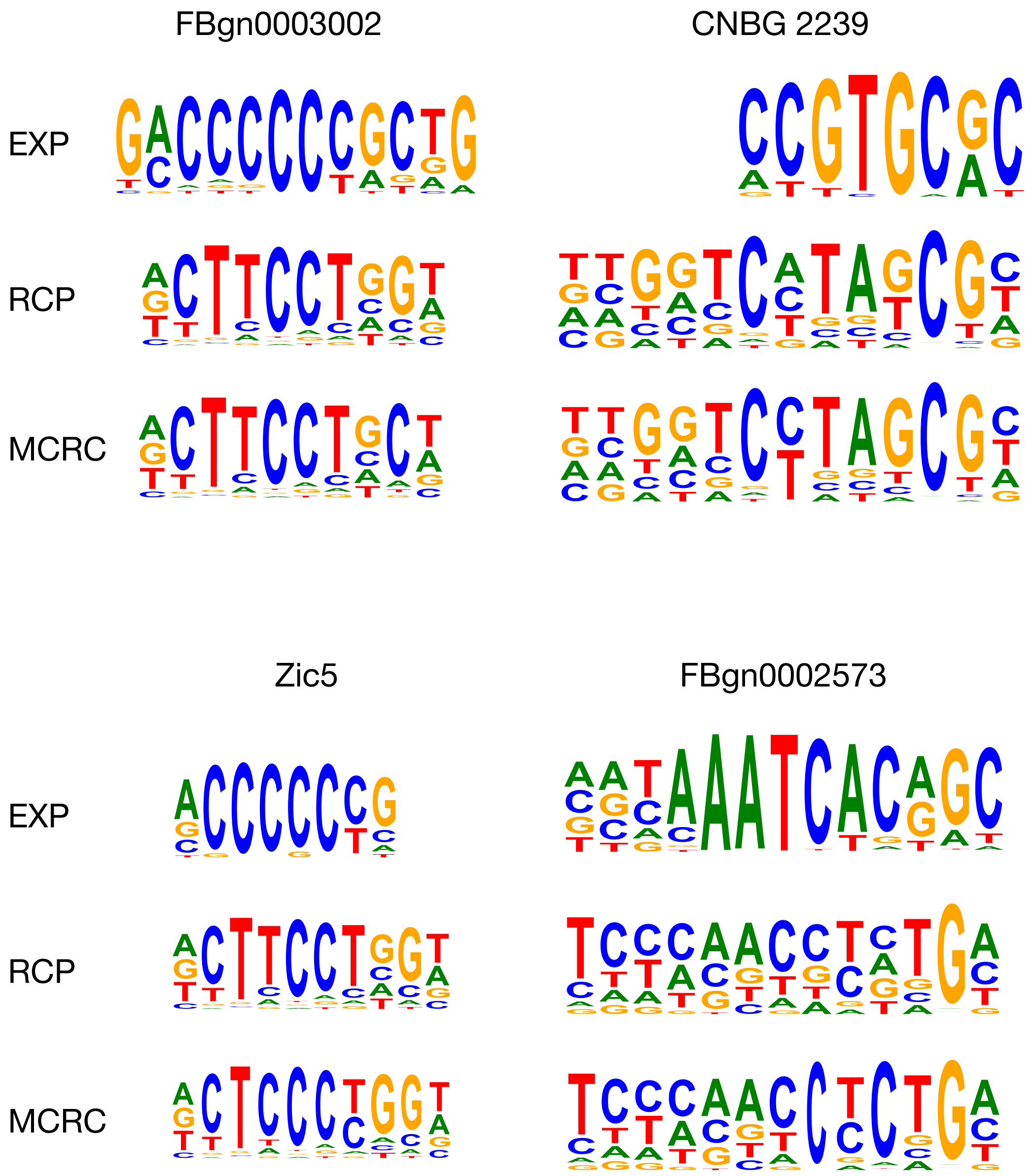

Figure S8. **Box plot showing the range of similarities between the best alignments of model motifs with HT-SELEX produced motifs.**

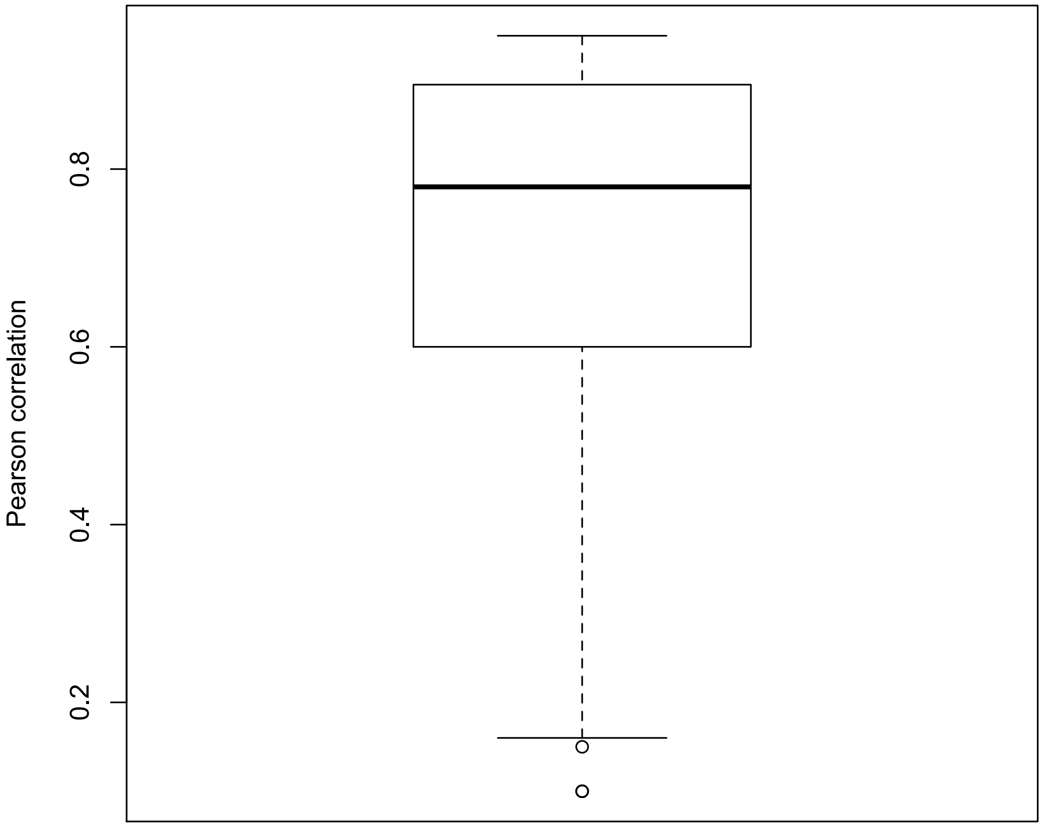

Supplement: SUPPLEMENTARY DATA [file supp_gkv919_nar-01494-z-2015-File008.pdf]
